# Accuracy Evaluation of GPT-Assisted Differential Diagnosis in Emergency Department

**DOI:** 10.3390/diagnostics14161779

**Published:** 2024-08-15

**Authors:** Fatemeh Shah-Mohammadi, Joseph Finkelstein

**Affiliations:** Department of Biomedical Informatics, School of Medicine, University of Utah, Salt Lake City, UT 84112, USA; fatemeh.shah-mohammadi@utah.edu

**Keywords:** emergency department, differential diagnosis, GPT, patient outcomes

## Abstract

In emergency department (ED) settings, rapid and precise diagnostic evaluations are critical to ensure better patient outcomes and efficient healthcare delivery. This study assesses the accuracy of differential diagnosis lists generated by the third-generation ChatGPT (ChatGPT-3.5) and the fourth-generation ChatGPT (ChatGPT-4) based on electronic health record notes recorded within the first 24 h of ED admission. These models process unstructured text to formulate a ranked list of potential diagnoses. The accuracy of these models was benchmarked against actual discharge diagnoses to evaluate their utility as diagnostic aids. Results indicated that both GPT-3.5 and GPT-4 reasonably accurately predicted diagnoses at the body system level, with GPT-4 slightly outperforming its predecessor. However, their performance at the more granular category level was inconsistent, often showing decreased precision. Notably, GPT-4 demonstrated improved accuracy in several critical categories that underscores its advanced capabilities in managing complex clinical scenarios.

## 1. Introduction

Diagnostic errors represent a major contributor to iatrogenic harm across all clinical settings including the emergency department (ED). Diagnostic errors surpass the combined morbidity and mortality associated with all other medical errors [1]. Annually, at least 12 million diagnostic errors occur in the United States, with numbers potentially being much higher [1,2]. Misdiagnosis-related harms, affecting between approximately 40,000 and 10 million Americans each year, range from minor to catastrophic, including permanent disability or death [1]. The economic burden of these errors on the U.S. healthcare system could surpass USD 100 billion annually [3,4].

Although diagnostic errors significantly impact patient safety and societal well-being, they often go unnoticed. Typically, these errors are not immediately evident; they usually surface later and are often identified by a different clinician or following incidents of misdiagnosis-related harm. This highlights the urgent need for enhanced diagnostic tools to support physicians in making accurate diagnoses [5]. Among the promising technologies being explored are clinical decision support (CDS) systems, including symptom checkers [6] and differential diagnosis generators [7]. Symptom checkers are designed primarily for the public, whereas differential diagnosis generators cater to healthcare professionals. The inception of computer-aided healthcare traces back to the early 1970s, marked by a strong interest in utilizing computational power to improve care quality. Historically, CDS tools have integrated multiple processes—logical or computational methods, probabilistic evaluations, and heuristic techniques—with many applications employing a combination of algorithms and heuristic rules [8,9]. Despite their potential to enhance diagnostic precision and efficiency, these systems often increase the workload for clinicians [10], largely due to the need for structured input data, which can hinder widespread adoption. In this landscape, artificial intelligence (AI) emerges as a viable alternative in providing healthcare support [11]. AI systems apply advanced algorithms, machine learning techniques, and statistical pattern recognition to process and analyze medical data. Unlike the rule-based counterparts, the modern AI-based systems are designed to evolve, continuously updating and refining their capabilities and outputs based on new data. The integration of AI based on deep learning into CDS systems is accelerating, underscoring the increasing reliance on sophisticated technologies in the healthcare sector. In particular, the advent of generative AI through Large Language Models (LLMs) has been transformative, significantly enhancing diagnostic accuracy, treatment planning, and overall patient care [12,13,14]. These AI systems mimic human cognitive processes and learn autonomously from continuous streams of new data. As they assimilate vast amounts of complex patient information, AI-enhanced CDS systems provide healthcare professionals with invaluable insights, thereby improving the efficacy of clinical decision making and leading to better patient outcomes. This ongoing evolution marks a significant shift in how healthcare systems leverage technology to meet the demands of modern medicine [15,16].

Among AI technologies, LLMs represent a sophisticated class of AI algorithms that have been meticulously trained on vast amounts of textual data. This training enables them to process and produce human-like text, which is proving invaluable in the field of medical diagnostics. The ability of these models to parse and synthesize complex information allows them to provide insights that were previously beyond the reach of automated systems. Tools like Google’s Bard (now Gemini) [17,18], Meta AI’s LLM Meta AI 2 (LLaMA2) [19], and OpenAI’s Chat Generative Pre-Trained Transformer (ChatGPT) [20] are prominent examples of such technologies now accessible to the public and the medical community alike. These generative AI tools are not only innovative due to their underlying technology, but also because of their demonstrated competence in practical applications [21]. Remarkably, models such as ChatGPT have been tested against rigorous standards such as national medical licensing examinations, where they have performed successfully without specific training tailored to these exams. This accomplishment underscores the potential of LLMs to significantly advance medical diagnostics. ChatGPT, in particular, has been the subject of extensive research within the healthcare sector, distinguishing itself as a leader in the application of generative AI [22,23].

Since its November 2022 launch, ChatGPT has rapidly gained significant attention, achieving over 100 million monthly active users within just two months of its release [24]. This success underscores the public’s engagement with the platform. GPT-3.5-turbo and its more advanced iteration, GPT-4, are LLMs that provide chat-based interaction capabilities, allowing for sophisticated responses to complex inquiries and problem-solving tasks [20]. Originally developed as versatile, general-purpose models, there is a growing interest in their applicability to specialized fields, including clinical settings. Preliminary investigations into their utility reveal that GPT-3.5-turbo can generate broadly suitable recommendations for simple cardiovascular disease prevention [25]. Furthermore, in the context of a public social media platform, GPT’s responses to health-related questions were not only favored but also perceived as more empathetic when compared to those from medical professionals [26]. Its efficacy has also been highlighted in studies where it has been used to respond to clinical vignette questions, showcasing its ability to operate as a powerful diagnostic tool [27]. The recent review [28] assessed the capability of ChatGPT to provide medical information on topics frequently discussed by patients with inflammatory bowel disease with their gastroenterologists. Various studies have evaluated how well these AI tools perform in generating differential diagnosis lists, a critical step in the diagnostic process [29,30,31]. While some studies cast a wide net by comparing multiple state-of-the-art models, our study is aimed at assessing the unique capabilities of the zero-shot prompting of ChatGPT-3.5 and ChatGPT-4 to generate differential diagnostic lists for patients admitted to the emergency department (ED) with the purpose of exploring their potential effectiveness as diagnostic aids.

## 2. Materials and Methods

### 2.1. Dataset

The main data source for analysis in this paper is the Medical Information Mart for Intensive Care III (MIMIC-III) dataset. This dataset is a widely utilized and comprehensive source of de-identified healthcare data. It contains detailed clinical information from over 60,000 critical care patients admitted to the Beth Israel Deaconess Medical Center (Boston, MA, USA) spanning a period of nearly a decade. This rich dataset includes electronic health records, lab results, prescription records, and clinical notes, making it a valuable resource for medical research, particularly in the fields of critical care, epidemiology, and health informatics. Within the dataset, our analysis was focused on patients who were admitted to the ED, and among all notes documented for these patients, we only used the notes that were documented within the first 24-hour period following admission to the ED. There were 17,971 unique patients admitted to the ED with 22,754 unique admissions. Of 17,971 unique patients, 2758 patients were admitted to the ED more than once. We considered 3000 random admissions.

### 2.2. Study Design

We assessed the diagnostic accuracy of differential diagnosis lists produced by ChatGPT-3.5 and ChatGPT-4. The term ‘differential diagnosis’ denotes a list of potential conditions or diseases that might explain a patient’s symptoms and signs, formulated based on the patient’s clinical history, physical examination, and any investigative results, thus facilitating the diagnostic process. We employed the ChatGPT-3.5 model (gpt-3.5-turbo model) and the ChatGPT-4 model (gpt-4). Neither model was specifically trained or enhanced for medical diagnosis. 

Clinical notes from the ED served as the basis for predictive modeling, while hospital discharge summaries documented the final diagnoses. These diagnoses are typically documented by responsible clinical staff at the point of care and reflect the consensus medical opinion at the time of patient discharge. The discharge diagnoses represent the final result of comprehensive patient review by a multi-disciplinary team of providers based on the entirety of diagnostic, laboratory, imaging, pathology, and any other relevant information collected throughout the patient hospital stay and reviewed by medical experts. Diagnosis codes within the MIMIC-III database are initially in ICD-9-CM format, which were converted to ICD-10-CM for standardization and contemporary relevance. These standardized ICD-10-CM codes were then utilized as the ground truth against which the GPT-predicted diagnoses were evaluated.

Engaging with LLMs necessitates the application of prompt engineering. Prompt engineering is the practice of crafting inputs or “prompts” for LLMs to effectively direct the model’s response towards a desired output. This technique is crucial because the quality of the input significantly influences the model’s output. Skilled prompt engineering enhances the precision and relevance of the responses from LLMs, making them more useful in practical applications such as content creation, or even complex decision-making scenarios. Effective prompts can reduce the number of iterations needed to reach an accurate or satisfactory answer, which leads to an increase in efficiency. Well-engineered prompts can exploit the full capabilities of an LLM, unlocking sophisticated behaviors and deeper insights from the model that are not immediately apparent through simple queries. Our final prompt selected was the following: “Using the patient text in the following, give, in bullets, the ranked list of most potential clinical diagnoses for this patient: <put the clinical text here>”. The clinical text that needs to be embedded in the prompt is the first-day-documented notes from ED admissions that were concatenated. The integration of these clinical notes into GPT’s framework allows the model to apply its advanced understanding of medical terminology and context to infer plausible medical conditions. This is achieved through the model’s ability to analyze the text for symptomatic mentions, historical medical information, and any initial treatments documented to be able to generate a differential diagnosis list. After prompting the GPT, it generated a ranked list of diagnoses, which was then converted into ICD-10-CM codes. These diagnoses were compared with actual discharge diagnoses. These codes, along with discharge diagnoses codes, were categorized using the Clinical Classifications Software Refined (CCSR) mapping algorithm from the Agency for Healthcare Research and Quality (AHRQ) [32]. The CCSR for ICD-10-CM diagnoses aggregates more than 70,000 ICD-10-CM diagnosis codes into clinically meaningful categories across 22 body systems, which generally follow the structure of the ICD-10-CM diagnosis chapters. The term “body system” is used to describe the organization of conditions within the CCSR tool. The CCSR categories are organized by body system. Each body system is abbreviated using a three-character scheme. Individual CCSR categories are numbered sequentially with the numbering scheme starting at “001” within each body system (i.e., there is a CCSR 001 for each body system). A complete listing of all CCSR categories and their associated descriptions can be found in the CCSR Reference File, available on the CCSR page [33]. For the analysis of body systems, the first three letters of the ‘CCSR CATEGORY 1’ column were extracted. All diagnoses were compared at two levels: the categorical level (over 530 categories) and the body system level (22 categories) [33].

### 2.3. Evaluation

We implemented two approaches to evaluate the diagnostic accuracy of ChatGPT-generated differential diagnoses. First, accuracy was assessed categorically by comparing each ChatGPT diagnosis with its corresponding final discharge diagnosis and categorizing the results into three scenarios. To do so, we first calculated the percentage of matched diagnoses using the formula below (*Dx* stands for diagnosis).

(1) $$\mathrm{Percent}\mathrm{of}\mathrm{matched}\mathrm{diagnosis}=\frac{number\;of\;matched\;Dx}{number\;of\;discharge\;Dx}\times 100\%$$

After calculating case match percentages for each visit, we classified them into three distinct levels: “no match” or “mismatch” (0%), “partial match” (>0% and <100%), and “full match” (100%). A “no match” was determined when none of the ChatGPT diagnoses aligned with the final discharge diagnoses, while a “full match” was determined when all ChatGPT diagnoses were explicitly diagnosed in the final hospital discharge diagnoses. A “partial match” was considered when only parts of the ChatGPT diagnoses were present in the hospital discharge diagnoses.

Second, diagnostic concordance was also evaluated on a systemic level. This involved aggregating the diagnoses into broader body system categories, as delineated by the CCSR. The accuracy for each body system was determined by the ratio of the number of GPT-predicted diagnoses that matched the body system of the discharge diagnoses to the total number of discharge diagnoses recorded for that same body system across all admissions.

## 3. Results

The analytical dataset comprised 3000 unique ED admissions. Patients on average had 15.87 ± 20.25 discharge diagnoses per admission. Patients’ concatenated first day ED notes on average consisted of 663.40 ± 613.88 GPT tokens. 

According to Table 1, at the body system level, GPT-3.5 and GPT-4 show a relatively similar frequency of ‘No match’ with diagnoses at 9.09% and 9.38%, respectively. The ‘Partial match’ category, which indicates the models’ ability to predict some but not all of the diagnoses, is where both models perform best. GPT-3.5 achieved a partial match 79.53% of the time, while GPT-4 showed a slight increase to 81.27%. However, for a ‘Complete match’, where the models’ predictions fully aligned with the actual diagnoses, both models scored low, with GPT-3.5 at 11.38% and GPT-4 slightly lower at 9.35%.

When assessing performance at the category level, the number of ‘No match’ results increases considerably for both models, with GPT-3.5 at 33.66% and GPT-4 at 36.01%, suggesting a more significant challenge in accurately predicting specific diagnosis categories. ‘Partial matches’ still constitute most of the predictions, with GPT-3.5 at 65.47% and GPT-4 at 63.37%. However, a ‘Complete match’ at the category level is notably rare, with GPT-3.5 achieving a mere 0.87% and GPT-4 at an even lower 0.62%.

**Figure 1:**
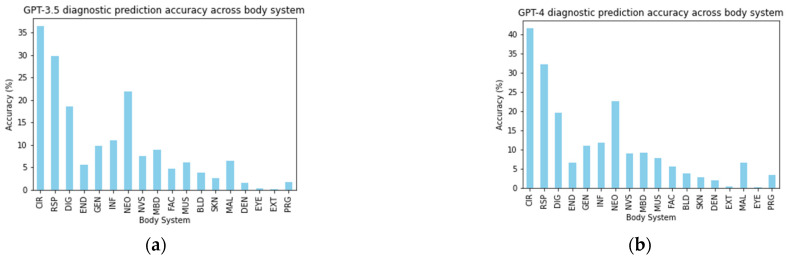
Comparative accuracy of GPT model predictions at the body system level: (**a**) performance of GPT-3.5; (**b**) performance of GPT-4.

Table 2 presents a comparative analysis of the diagnostic accuracy of GPT-3.5 and GPT-4 against actual discharge diagnoses across various body system categories using the CCSR. Each row represents a different body system, with the table organized by the frequency (‘*n*’) of correct predictions made by each GPT model and the total number of corresponding discharge diagnoses for each system. The columns labeled “GPT-4” and “GPT-3.5” show the counts of accurate predictions across all admissions by the respective models, while the “Discharge Dx” column reflects the total counts of diagnoses documented at discharge for each body system. Figure 1 provides a visual representation of the diagnostic accuracy of the GPT-3.5 and GPT-4 models across various body system categories, as detailed in Table 2. The *y*-axis in the sub-figures represents the ratio of the number of correct diagnoses predicted by the GPT models (GPT-3.5 and GPT-4) to the total number of discharge diagnoses within the same body system, as recorded in Table 2.

The data exhibited in Table 2 and Figure 1 underscore the comparative efficacy between GPT-4 and GPT-3.5 across various body systems. Both GPT models demonstrated the highest predictive accuracy for ‘Diseases of the circulatory system (CIR)’, with GPT-4 surpassing GPT-3.5, yielding 4017 correct predictions compared to 3523, against 9681 actual discharge diagnoses in this category. This suggests a more refined ability of GPT-4 to contextualize and analyze symptoms pertinent to circulatory conditions. Moreover, a substantial number of correct predictions were made by both models in ‘Diseases of the respiratory system (RSP)’, with GPT-4 once again achieving higher accuracy with 1223 correct predictions compared to GPT-3.5’s 1127, out of 3791 discharge diagnoses. Another area where both models performed relatively well is in ‘Diseases of the digestive system (DIG)’, although with a lower number of correct predictions than the aforementioned systems, standing at 601 for GPT-4 and 571 for GPT-3.5 out of a total of 3090 discharge diagnoses.

In contrast, body systems like ‘Endocrine, nutritional, and metabolic diseases (END)’ and ‘Diseases of the blood and blood-forming organs and certain disorders involving the immune mechanism (BLD)’ reflected a notable decline in the number of correct predictions, particularly when considering the total number of discharge diagnoses present in the dataset for these categories. Furthermore, the ‘Mental, behavioral, and neurodevelopmental disease (MBD)’ and ‘Diseases of the musculoskeletal system and connective tissue (MUS)’ also observed fewer correct predictions. Figure 1, illustrating the accuracy of predictions at the body system level for both GPT models, visually confirms the findings in Table 2.

**Figure 2:**
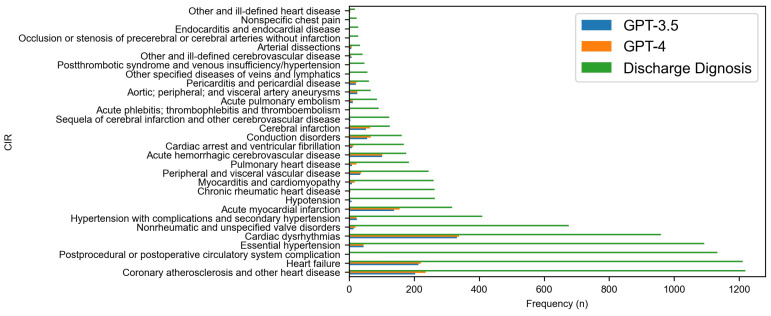
Comparative accuracy of GPT model predictions at category level for CIR body systems.

**Figure 3:**
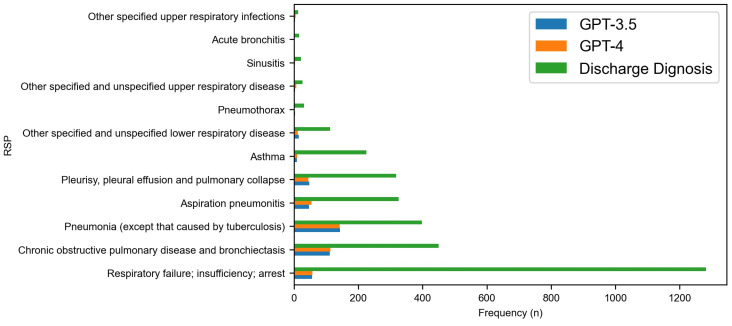
Comparative accuracy of GPT model predictions at category level for RSP body systems.

**Figure 4:**
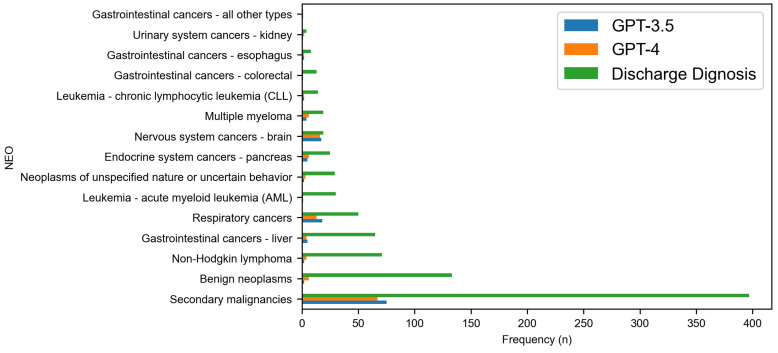
Comparative accuracy of GPT model predictions at category level for NEO body system.

**Figure 5:**
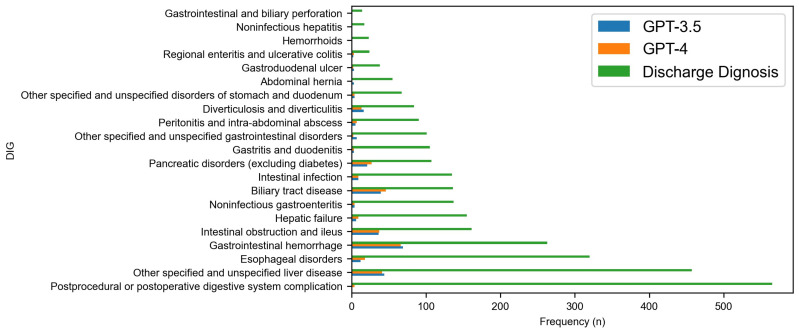
Comparative accuracy of GPT model predictions at category level for DIG body system.

Figure 2, Figure 3, Figure 4 and Figure 5 delve deeper into the four body systems, where high accuracy was initially observed. These figures generally depict the frequency or prevalence of various medical conditions at the time of hospital discharge, providing a baseline for comparative analysis against model predictions. Additionally, they illustrate the frequency of accurate diagnoses made by the GPT-3.5 and GPT-4 models. Figure 2 and Figure 3 in particular delve into ‘Diseases of the circulatory system (CIR)’ and ‘Diseases of the respiratory system (RSP)’ body systems. For CIR, in the initial part of the chart, where discharge diagnoses are more frequent, both models exhibit relatively high accuracy, suggesting that these conditions, being more common, possibly have clearer diagnostic markers that are well-captured by the models. Notably, GPT-4 generally shows a higher accuracy than GPT-3.5, indicative of its enhanced modeling capabilities. However, as the conditions become less frequent towards the right side of the chart, a marked decrease in the models’ accuracy is observed. This decline in performance might reflect the increased difficulty in diagnosing less common diseases that manifest with subtler or less distinct symptoms.

The detailed results in Figure 3 for the ‘Diseases of the respiratory system (RSP)’ body system level exhibit a differential performance across various respiratory conditions, which provides an insight into the relative strengths of each model in parsing and analyzing specific respiratory-related clinical data. In the case of ‘Pneumonia’, a prevalent condition, both GPT models demonstrate moderately high diagnostic accuracy, aligning closely with the higher frequency of discharge diagnoses. Specifically, GPT-4 shows a marked improvement in diagnostic accuracy for ‘Chronic obstructive pulmonary disease and bronchiectasis’, surpassing GPT-3.5 slightly, suggesting refined capabilities in identifying this condition. Notably, for ‘Acute bronchitis’, both models exhibit low diagnostic accuracy, which does not proportionally reflect the moderate frequency of this diagnosis at discharge, indicating a potential area for model improvement. Overall, the data reveal that, while both models perform well in identifying common respiratory diseases, there remains room for enhancement, especially in conditions with lower discharge frequencies.

Figure 4 and Figure 5 delve deeper into the ‘Neoplasms (NEO)’ and ‘Diseases of digestive system (DIG)’ body systems. Regarding the NEO body system, with a focus on those that exhibit a higher frequency of discharge diagnoses, the category of ‘Nervous System Cancers—Brain’ not only shows a high frequency of discharge diagnoses, but also demonstrates a high accuracy rate of 89.47% with GPT-3.5, although there is a slight decrease to 84.21% with GPT-4. This category is significant due to both the high frequency of cases and the high accuracy observed, particularly with the earlier model iteration. Another key observation is in ‘Respiratory Cancers’, where, despite a high frequency of diagnoses, there is a notable decrease in diagnostic accuracy from GPT-3.5 at 36.00% to GPT-4 at 26.00%, indicating potential areas for improvement in the newer model. Additionally, ‘Urinary System Cancers—Kidney’ shows a significant improvement in accuracy from GPT-3.5 at 25.00% to GPT-4 at 50.00%, reflecting robust enhancements in GPT-4’s diagnostic capabilities.

The analysis of differential diagnosis accuracy for diseases of the digestive system (DIG) by the GPT-3.5 and GPT-4 models reveals notable trends and highlights significant categories. The frequency of discharge diagnoses indicates that certain categories within the digestive system diseases were more prevalent than others. Focusing on categories with higher discharge diagnosis frequencies provides a more relevant insight into the performance of the models. The graph indicates a notable decline in the frequency of discharge diagnoses from left to right, with the most common conditions appearing towards the left side of the graph. Both GPT-3.5 and GPT-4 show higher diagnostic accuracy for the more frequently diagnosed conditions, suggesting that both models perform better with conditions that are more common or perhaps more distinct in their presentation.

For the conditions with the highest discharge frequencies, GPT-4 generally matches or slightly surpasses the performance of GPT-3.5, indicating incremental improvements in model capabilities from one generation to the next. However, for conditions with lower discharge frequencies, the accuracy of both models diminishes, which may reflect the challenges AI models face when dealing with less common or more complex diagnostic scenarios.

In diseases such as ‘Gastroesophageal reflux disease (GERD)’ and ‘Gallbladder disease’, both models demonstrated relatively higher diagnostic accuracy. GPT-4 consistently outperformed GPT-3.5, aligning with the general trend of improved capabilities in later model iterations. This suggests that GPT-4 may be better at identifying symptoms and correlating them with specific conditions within the digestive system. Categories with lower diagnostic accuracy across both models, and which also had significant discharge diagnosis frequencies, included peptic ulcer disease and acute pancreatitis. In these cases, both models struggled to reach a high level of accuracy, which might indicate the complexity and symptom overlap common in these conditions that pose challenges for AI-driven diagnostic tools. For categories with low discharge frequencies, such as chronic pancreatitis and liver diseases, the performance of the models was not as critical from a clinical utility perspective due to the rare nature of these conditions in the dataset. However, where GPT-4 did engage with these rarer conditions, it showed a marginal improvement over GPT-3.5, suggesting incremental advancements in handling fewer common diseases within the digestive system.

## 4. Discussion

In this study, the accuracy of differential diagnosis lists generated by ChatGPT-3.5 and ChatGPT-4 using the clinical notes recorded during the first 24 h of ED admission was assessed. These models processed unstructured text to formulate a ranked list of potential diagnoses. The accuracy of these models was benchmarked against actual discharge diagnoses to evaluate their utility as diagnostic aids. The results in Table 1 and Table 2 indicate that, while both models are reasonably effective at identifying some correct diagnoses within broader body systems, their performance diminishes significantly when the task is narrowed down to specific categories. 

Given the higher ‘Partial match’ rate denoted in Table 1, GPT-4 appears to slightly outperform GPT-3.5 in overall diagnostic prediction at the body system level. According to Table 2 and Figure 1, GPT-4 also outperformed GPT-3.5 in predicting the correct discharge diagnoses. This was observed across most body systems, with the diseases of the circulatory system presenting the highest number of correct predictions by both models. The disparity between the models’ performances and the actual discharge diagnoses highlights potential areas for model refinement. In the circulatory system, certain conditions like ‘Acute myocardial infarction’ and ‘Acute hemorrhagic cerebrovascular disease’ showed relatively high accuracy rates for both GPT models, with GPT-4 generally outperforming GPT-3.5. This suggests that GPT-4’s enhancements in model architecture might be better at interpreting the complex clinical features of severe cardiovascular conditions. Conversely, both models struggled with less common conditions such as ‘Hypotension’ and ‘Postprocedural or postoperative circulatory system complications’, which had low accuracy, indicating difficulties in diagnosing less distinct or infrequent conditions. In conditions with low discharge frequencies like ‘Asthma’ and ‘Respiratory failure; insufficiency; arrest’, both models showed lower accuracy, underscoring potential challenges in capturing the complexities associated with such conditions through AI analysis. It is evident that, while the models can identify common respiratory diseases relatively well, they struggle with less common or more complex conditions.

Overall, across all body systems, our results consistently show that, while GPT-4 tends to outperform GPT-3.5 in most categories, particularly in those involving complex and severe conditions, both models still show room for improvement in less common diseases. The higher frequency of certain conditions at discharge generally correlates with better model performance, suggesting that more common conditions are better represented in the training data, thus making it easier for the models to learn and predict accurately. However, the accuracy diminishes for conditions that are infrequent or have subtler clinical presentations, highlighting an area where future model training could focus on improving. These findings point towards the continued evolution of AI models in medical diagnostics and the need for ongoing advancements to enhance their precision and reliability in clinical applications. In these instances, despite the technological advances embedded in these AI models, the challenge of accurately diagnosing rare conditions remains evident. This pattern underlines the importance of continuous model training and refinement, particularly focusing on less common conditions to potentially improve diagnostic outcomes in clinical settings.

Our results demonstrate the significant potential of LLMs in reducing diagnostic discrepancy, which is especially important in the fast-paced ED setting. In emergency medicine, diagnostic discrepancy is defined as the difference between the diagnosis made by the emergency department physicians and the final diagnosis made by the hospitalists or specialists after the patient has been admitted to the hospital [34]. Diagnostic discrepancy is a well-described phenomenon in healthcare [35,36], and it is common, especially in patients hospitalized via the ED [37,38]. According to previous reports, diagnostic error rates in patients admitted to the ED vary between 0.6% and 64% [39,40]. The variation in the reported error rates, at least in part, is explained by differences in how the diagnostic error was defined, and whether primary diagnoses, all diagnoses, missed diagnoses, or unintentionally delayed diagnoses were used in calculating diagnostic discrepancy [41,42,43]. Diagnostic discrepancy in the ED may result in serious patient harm [35,36] and, in certain instances, may be associated with heightened morbidity and mortality [38]. Diagnostic discrepancies can have significant implications, both for patients and healthcare providers, such as delayed treatment, increased healthcare costs, patient anxiety, distress and continued suffering, decreased patient trust, inappropriate treatment, overuse of resources, unnecessary or excessive referrals and consultations, negative impact on clinical decision making, and unnecessary treatments [44,45,46,47]. The Comparative Effectiveness Review of Diagnostic Errors in the ED, published in 2022 and based on the analysis of 279 studies, concluded that diagnostic discrepancies in the ED represent a significant patient safety challenge and that future research should emphasize areas in which the use of EHR data can facilitate differential diagnostics and a reduction in diagnostic uncertainty [48].

Our results are concordant with the recent findings on the use of LLMs to facilitate differential diagnostics [49,50]. Large Language Models (LLMs) are the most recent advancements in deep learning and present tremendous potential for AI applications in clinical care [51]. Language models such as Bidirectional Encoder Representations from Transformers (BERT) and Generative Pre-trained Transformers (GPT) can perform complex tasks such as text classification, question answering, named entity recognition, and text summarization and classification, which have the potential to augment clinical decision making in providing a differential diagnosis and reducing diagnostic uncertainty [52,53,54,55]. Recent studies reported the high accuracy of GPT-4 in complex diagnostic challenges [56] and generating differential diagnoses early in ED presentations with 98% accuracy [57]. Another study demonstrated that GPT-4 may increase confidence in diagnosis and earlier commencement of appropriate treatment, alert clinicians about missing important diagnoses, and offer suggestions similar to specialists to achieve the correct clinical diagnosis [58]. Similar results were reported by the application of pre-training and fine-tuning of existing BERT models [59,60,61].

As the primary goal of ED care is stabilization of the main organ systems of patients who are acutely ill or injured, comprehensive diagnostic work-up may be affected by the life-saving priorities of the fast-paced ED settings [62]. Previous studies showed that diagnostic delays in ED result in longer hospital stays and higher mortality [63]. Diagnostic tools that facilitate differential diagnostics may reduce diagnostic uncertainty and help arrive at the correct diagnosis earlier [64,65]. Thus, the further exploration of LLMs’ capabilities in facilitating differential diagnostic processes in ED is warranted.

### Limitations

Despite the demonstrated capabilities of ChatGPT-3.5 and ChatGPT-4 in enhancing diagnostic accuracy, several limitations must be addressed to ensure their effective integration into clinical settings. One significant limitation is the inconsistency in performance across different diagnostic categories, which can lead to varied reliability in their predictive outputs. While GPT-4 shows improved accuracy in critical diagnostic categories, its performance still varies, especially in more complex or less common conditions, which could undermine its utility in scenarios requiring consistent precision. Moreover, these models depend heavily on the quality and scope of the data they are trained on, limiting their effectiveness in scenarios where patient presentations are atypical or poorly documented in training datasets.

Furthermore, the current evaluation of these models does not fully account for the nuances of real-time clinical decision making, which often involves variables that extend beyond the textual data available in electronic health records. Factors such as patient-specific nuances, practitioner expertise, and interdisciplinary inputs, which are crucial in real-world settings, remain outside the scope of these models. This limitation highlights the need for a hybrid approach that combines AI insights with human oversight. Future applications must focus on creating more adaptable, context-aware systems that can function alongside healthcare professionals, providing support that enhances rather than replaces human judgment, ensuring a balanced integration of technology in healthcare practices.

## 5. Conclusions

Emergency department (ED) settings necessitate rapid and precise diagnostic evaluations to optimize patient outcomes and streamline healthcare delivery. This study assesses the effectiveness of generative pre-trained transformers, specifically the third-generation GPT-3.5 and fourth-generation GPT-4, in analyzing electronic health record notes from the initial 24 h following ED admission. These models were employed to process unstructured text, generating a hierarchy of potential differential diagnoses, which were subsequently evaluated against actual discharge diagnoses to determine the models’ diagnostic precision and their practical utility as diagnostic aids. The results demonstrate that both GPT-3.5 and GPT-4 hold considerable promise for aiding the early diagnostic process, showcasing commendable accuracy in generating partially correct matches at the body system level. Nonetheless, the study reveals limitations at the more detailed category level, where both models struggled to achieve the precision necessary for fully accurate diagnoses. This discrepancy highlights the need for ongoing improvements in AI technology to refine its capacity to discern and interpret the nuanced details essential for accurate medical diagnosis. While AI exhibits potential as a supportive tool in ED settings for formulating differential diagnoses, its integration into routine clinical practice requires continuous technological enhancements and rigorous clinical validation.

## Figures and Tables

**Table 1:** Differential diagnostic accuracy across GPT-3.5 and GPT-4 models.

	Body System		Category	
	GPT-3.5	GPT-4	GPT-3.5	GPT-4
	Percentage (%)	Percentage (%)	Percentage (%)	Percentage (%)
**No match**	9.09%	9.38%	33.66%	36.01%
**Partial match**	79.53%	81.27%	65.47%	63.37%
**Complete match**	11.38%	9.35%	0.87%	0.62%

No match, partial match, and complete match represent diagnostic match of 0%, higher than 0% and less than 100%, and 100%, respectively.

**Table 2:** Differential diagnostic accuracy across GPT-3.5 and GPT-4 models in body system level.

	GPT-4	GPT-3.5	Discharge Dx
Body System	*n*	*n*	*n*
Diseases of the circulatory system (CIR)	4017	3523	9681
Diseases of the respiratory system (RSP)	1223	1127	3791
Diseases of the digestive system (DIG)	601	571	3090
Endocrine, nutritional, and metabolic diseases (END)	330	282	5076
Diseases of the genitourinary system (GEN)	269	239	2449
Certain infectious and parasitic diseases (INF)	245	229	2077
Neoplasms (NEO)	221	216	983
Diseases of the nervous system (NVS)	158	133	1767
Mental, behavioral, and neurodevelopmental disease (MBD)	133	130	1465
Diseases of the musculoskeletal system and connective tissue (MUS)	107	90	1371
Factors influencing health status and contact with health services (FAC)	106	84	1943
Diseases of the blood and blood-forming organs and certain disorders involving the immune mechanism (BLD)	80	80	2131
Diseases of the skin and subcutaneous tissue (SKN)	37	35	1372
Dental diseases (DEN)	6	5	315
External causes of morbidity (EXT)	5	5	1802
Congenital malformations, deformations, and chromosomal abnormalities (MAL)	5	4	77
Diseases of the eye and adnexa (EYE)	3	2	1971
Pregnancy, childbirth, and the puerperium (PRG)	2	1	59

## Data Availability

There are no additional data deposited on any other site other than in this manuscript.
